# The Book World of Medicine and Science

**Published:** 1897-10-09

**Authors:** 


### The Book World of Medicine and Science.

The Spas of Wales. By T. R. Roberts. (London, 1897: John Hogg, 13, Paternoster Row. Pp. 120. Price 1s.)

This is an interesting little book, and gives the reader a very fair idea of the principal spas and mineral waters of the Principality. The author lays appropriate stress upon the advantageous circumstances under which the drugs they contain are administered, although he is perhaps a little too enthusiastic as to the therapeutic value of a mineral water as a solvent for iron. The principal wells described are those of Builth, Carnarvon, Llandrindod, Llangammarch, Llanwrtyd, and Trefriw, and there is a supplementary chapter referring briefly to a large number of miscellaneous springs. Not only are the constitution and properties of the springs fully treated, but a pleasant and often graphic account is given of their environment. The book, although obviously written by a layman, is singularly free from hyperbole as regards the curative powers of the different waters. Some of the analyses quoted are, it must be confessed, a little archaic, but, after all, the medicinal value of a spring can seldom be accurately deduced from its chemical constitution. The usual plan is the inverse of this; a water is found to have certain properties, and the results of analysis are more or less forcibly dragged into line with them. It must appear curious to the medical reader of to-day to see mention of "Dr. A. B. Garrod, physician to the University College Hospital," and statements are quoted as regards the Nauheim treatment of heart disease which betoken the compiler's lack of knowledge as to the authority with which their authors are entitled to speak. These are, however, but minor defects, and the book will form a pleasant companion during the long and rather tedious journey to the resorts of which it treats.

Anatomical Atlas of Obstetric Diagnosis and Treatment. By Oscar Schaeffer, M.D. With 145 illustrations. Hand Atlas Series, Vol. III. (London: Baillière, Tindall, and Cox. 1897. Price 12s. 6d.)

Atlas and Essentials of Gynæcology. By Dr. Oscar Schaeffer. With 173 coloured-plate illustrations and 54 wood-cuts. (London: Baillière, Tindall, and Cox. 1897. Price 15s.)

Every demonstrator knows how great are the advantages of good illustrations and how important, both as an aid to clearness of exposition and as a means of fixing facts in the memory, is the use of colours. Even though they be nothing more than the coloured chalks of the class-room they serve to impress upon the mind differences in the quality and the functions of the parts represented in a way that could not be otherwise attained. It is in this that the illustrations which form the great feature of the volumes before us, and, in fact, give to them the name of the Hand Atlas Series, are likely to be of such help to the student. The artists have evidently not tied themselves down to the manufacture of a coloured atlas. What they have tried to do is to make an explanatory atlas, using colour as a means of making clear at first sight what might otherwise have required much explanation, and there can be no doubt that in this they have succeeded. Some of the pictures are strikingly good, and many of the schematic drawings, perhaps partly from the absence of confusing detail, certainly present the points which they are intended to emphasise in a very definite and convincing manner. The plan of both these volumes is the same, the first half of each being occupied by the plates and the descriptions which are opposite each, and the second half being devoted to a systematic treatment of the subject.

The volume on obstetrics treats of that science primarily as a problem in anatomy and physiology. The anatomy of the pelvis, the relations of the fœtus, the nature of pelvic deformities and their influence on parturition, and the effects of displacements of the uterus and of the presence of tumours in retarding or preventing its completion are well described. The abnormalities which may be met with are, by aid of the illustrations, made very clear, and a study of the subject of extra-uterine pregnancy is very well presented.

The descriptions of surgical procedures seem hardly sufficiently full. Possibly it is considered that these things can only be learned from ocular demonstration, and, of course, when midwifery is taught as surgery is now taught, this criticism will fall to the ground. As things are at present, however, a text-book can hardly be too minute in describing the exact steps in obstetric operations, for many a practitioner finds himself called upon to do operations which he has had no opportunity of seeing as a student. For this reason we do not think that this atlas will take the place of the ordinary English manuals; in fact, it does not pretend to do so, but that every student will be the better for a careful study of its contents we are sure.

The volume on gynæcology gives an admirable review of the subject. Of course it is impossible to deal fully with the whole of gynæcology in a couple of hundred pages, but by taking the text and the illustrations together much may be learned, and we would go so far as to say that, by a careful study of the pictures and a reference to such parts of the text as is necessary to explain them, the student will gain a great deal of information which he is very apt to slur over in reading ordinary books. But here again the same criticism applies, and we think that the proper place of both these volumes is not as ordinary handbooks, but as a means of presenting that view of the subjects dealt with which one would get from studying them in a museum, and in doing this they certainly are very successful.

### BOOKS RECEIVED.

J. and A. Churchill.

"Elements of Human Physiology." By Ernest H. Starling, M.D., F.R.C.P.

Baillière, Tindall, and Cox.

